# Corpora Amylacea in Neurodegenerative Diseases: Cause or Effect?

**DOI:** 10.23937/2378-3001/2/2/1031

**Published:** 2015-08-28

**Authors:** Troy T. Rohn

**Affiliations:** Department of Biological Sciences, Boise State University, Boise, USA

**Keywords:** Corpora amylacea, Neurodegenerative disease, Vascular dementia, Pathology, Alzheimer’s disease

## Abstract

The presence of corpora amylacea (CA) in the CNS is associated with both normal aging and neurodegenerative conditions including Alzheimer’s disease (AD) and vascular dementia (VaD). CA are spherical bodies ranging in diameter (10–50 μm) and whose origin has been documented to be derived from both neural and glial sources. CA are reported to be primarily composed of glucose polymers, but approximately 4% of the total weight of CA is consistently composed of protein. CA are typically localized in the subpial, periventricular and perivascular regions within the CNS. The presence of CA in VaD has recently been documented and of interest was the localization of CA within the hippocampus proper. Despite numerous efforts, the precise role of CA in normal aging or disease is not known. The purpose of this mini review is to highlight the potential function of CA in various neurodegenerative disorders with an emphasis on the potential role if any these structures may play in the etiology of these diseases.

## Characteristics of Corpora Amylacea

**Figure 1:**
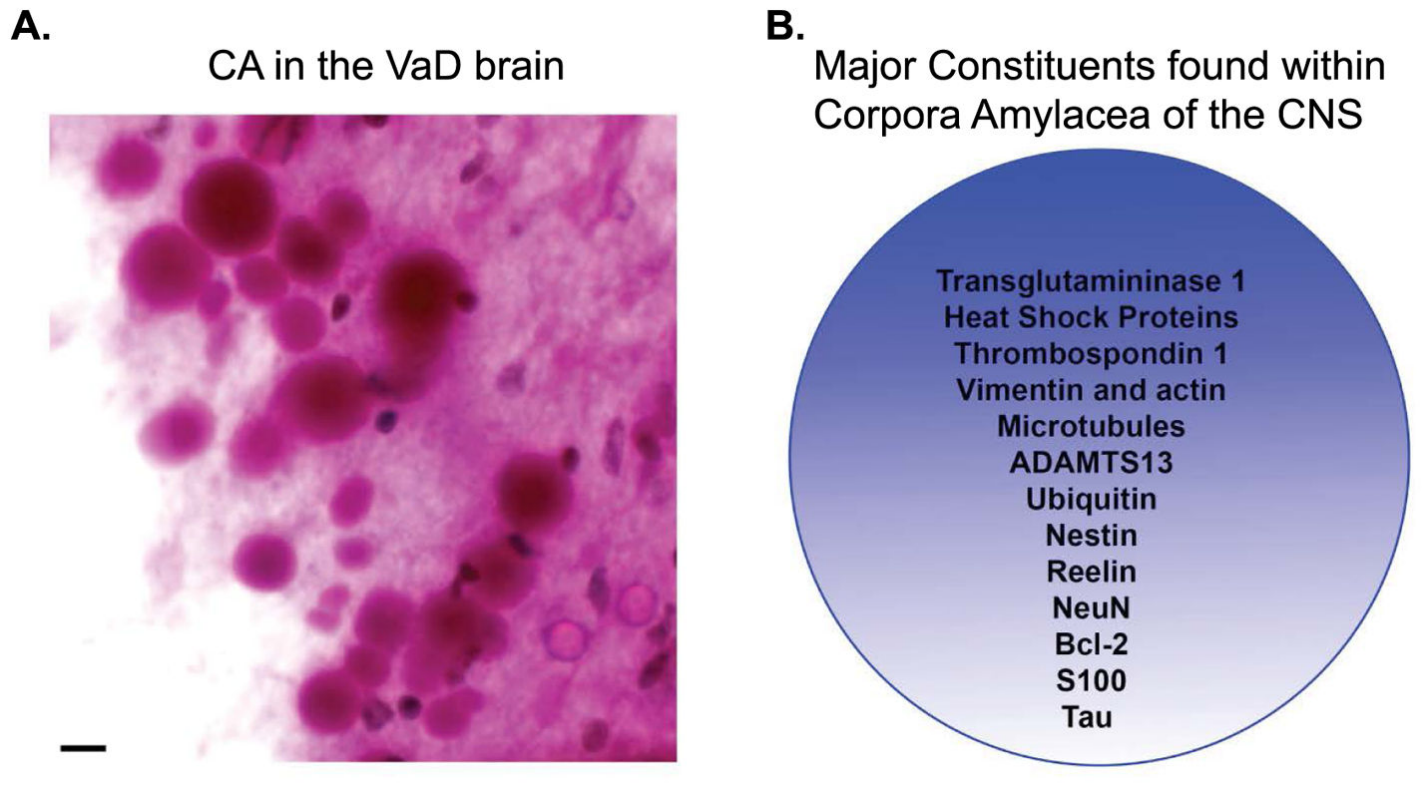
Characteristic features of corpora amylacea in the CNS **(A):** CA are basophilic structures principally composed of polysaccharides and as such are easily identified using periodic acid-Schiff (PAS) reagent that labels CA as pink circular structures with varying diameters between 10–50μm. Depicted are numerous CA detected following labeling with PAS reagent in subpial regions in the hippocampus of a representative VaD case. Scale bar represents 10μm. **(B):** The major protein constituents that have been documented within CA are numerous and include both cytoskeletal proteins, stress proteins, and blood proteins.

Corpora amylacea (CA) were first described by Purkinje in 1837 in the brains of elderly patients. The presence of CA within the normal aging brain is well established and in addition, can also be seen in a variety of neurological conditions including Alzheimer’s disease (AD), multiple sclerosis, hippocampal sclerosis and epilepsy [1–6]. Morphologically, CA represent as spherical translucent structures that range in diameter between 10–50μm with an average diameter of 15 μm [7,8]. Their internal structure is characterized by the presence of numerous short linear densities with a narrow rim of fibrils often seen at the periphery. Histochemical analysis indicates that CA are basophilic structures principally composed of polysaccharides and as such are easily identified using periodic acid-Schiff reagent or iodide (Figure 1A). In addition to polysaccharides, numerous proteins involved in aging and stress have been identified within CA including ubiquitin, heat-shock proteins [9], transglutaminases [10], anion exchange proteins [11], complement proteins [12], myelin basic protein [13], Bcl-2, c-Jun [14], NeuN [15], S100 proteins [16], thrombospondin-1, ADAMTS13 [17], reelin [18], tau [18–20], and alpha-synuclein [18] (Figure 1B).

Much debate has centered around the potential origin of CA within the CNS as either being derived from glial or neuronal cells. Historically, CA were interpreted as being glial in nature [7,21,22]. On the other hand, evidenced has suggested that CA are derived from neuronal sources [13,19,23–27]. It is noteworthy, that a recent study has provided compelling evidence that CA are derived from a glial source that involves heme oxygenase-1-mediated damage to mitochondria and CA biogenesis [28]. The authors came to these conclusions by using cultured astrocytes in which heme oxygenase-1 was over expressed to levels typically observed in the AD brain. CA-like cytoplasmic inclusions were consistently observed under these conditions within astrocytes that were not present in control cells [28].

With advancing age, CA are frequently found in the sub ependymal zones of the ventricles, localized in particular along the margin of blood vessels or beneath the pia [7]. The prevailing view suggests that because CA develop in the aged individuals with documented vascular disease and diabetes, that disturbances in the blood-brain barrier may underlie why CA develop mainly in the proximity of structures composing this barrier including the perivascular space, subpial and subependymal areas [29].

## Presence of Corpora Amylacea in Neurodegenerative Diseases

CA accumulation in the CNS is associated with a number of neurodegenerative diseases. In AD, CA are present to a greater density as compared to normal aged subjects and can be identified by ubiquitin, tau, and heat-shock protein antibodies [9,19]. Renkawek and Bosman reported the presence of anion exchanger proteins within CA of the AD brain and their data supported an accumulation of neuronal proteins may be involved in the pathogenesis of CA in AD [11]. Although CA documented from both the brain of normal aging individuals versus AD showed morphological similarities, there have been reported differences, for example, in their size, biochemical and elemental composition [19].

CA have also been documented in hippocampal sclerosis and temporal lobe epilepsy [3–5,30–33]. In contrast to AD, CA in hippocampal sclerosis and temporal lobe epilepsy appear to localize predominantly within the hippocampal proper and not in perivascular regions. In addition, the distribution of CA parallels the neuronal cell loss associated with hippocampal sclerosis and the inverse correlation of CA density with neuronal cell densities suggests that CA may be the result of neuronal cell loss [4,5,34]. In a study by Cherian et al., the authors examined the role of CA in patients with mesial lobe epilepsy associated with temporal sclerosis. Their findings demonstrated that patients with the presence of CA in their hippocampi were significantly older and showed a trend towards having a longer duration of epilepsy as compared to patients who did not have CA [33].

In addition to AD, hippocampal sclerosis and temporal lobe epilepsy, CA have also been documented in other neurodegenerative diseases including multiple sclerosis [2,35], Parkinson’s disease [10,36,37], Huntington’s disease [38], and Pick’s disease[12]. With regards to the finding of CA in multiple sclerosis, the authors concluded that the generation of CA are most likely a secondary phenomenon in the pathology of this disease [2]. Moreover, Selmaj et al. demonstrated that CA represent remnants of degenerated and aggregated neuronal cells [2].

## Presence of Corpora Amylacea in Vascular Dementia

Vascular dementia (VaD) is a neurodegenerative disorder that accounts for roughly 15–20 percent of all types of dementia making it the second leading cause of dementia behind only AD in the USA [39]. Available data indicates that VaD shares several pathological features with AD, including the presence of neurofibrillary tangles (NFTs), amyloid or plaques, white matter lesions and cerebral amyloid angiopathy [40,41]. According to a recent analysis, pure vascular dementia (VaD) accounts for roughly 15–20 percent of all types of dementia making it the second leading cause of dementia behind only Alzheimer’s disease in the USA [39]. One difficulty in measuring the prevalence of VaD is that it often coexists with Alzheimer-type lesions and other pathologies with 20–30 percent of demented subjects showing mixed pathologies [42]. Indeed, available data indicates that VaD and AD share several pathological features including the presence of neurofibrillary tangles (NFTs), amyloid or plaques, white matter lesions and cerebral amyloid angiopathy [40,41]. When VaD has concomitant AD pathology the symptoms are collectively referred to as being of “mixed dementia”. Behaviorally, patients with VaD show loss in executive functions as an initial symptom, whereas in AD memory loss is often associated with the earliest known symptoms. Other important symptoms of VaD include confusion, language deficits, restlessness, agitation, and gait disturbances [43]. There are at least three pathological features commonly associated with VaD that include: 1) large artery infarctions, 2) small artery infarctions or lacunes that are generally subcortical, and 3) chronic subcortical ischemia leading to selective loss of neurons, glial cells, and endothelial cells [44].

**Figure 2:**
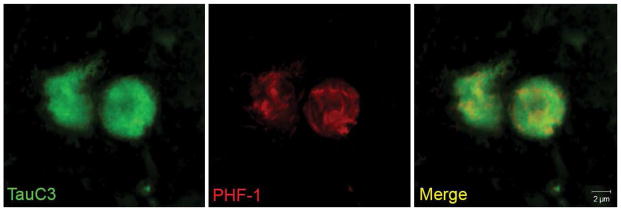
Co-localization of caspase-cleaved tau with PHF-1 within corpora amylacea of the VaD brain Representative images from double-label immunofluorescence confocal analysis in VaD utilizing the TauC3 antibody that detects caspase-cleaved tau (green, far left panel) and PHF-1 (red, middle panel) with the overlap image shown indicating co-localization of the two markers (yellow, far right panel). The results revealed a fibrillar labeling by PHF-1 while that of TauC3 was more or less homogenous throughout CA.

We have recently demonstrated the presence of caspase-cleaved tau in the human VaD brain [45]. This study was accomplished utilizing a well-characterized antibody, TauC3 that detects caspase-cleaved tau truncated at Asp^421^ [46,47]. Using the TauC3 antibody, we showed the presence of caspase-cleaved tau within NFTs, neuropil threads, and CA. The presence of caspase-cleaved tau within CA was a consistent finding in all VaD cases examined [45]. Importantly, labeled CA were abundantly found only in the hippocampal proper, specifically within the dentate gyrus region. In addition, we were able to document the co-localization of TauC3 together with PHF-1, an antibody that detects paired-helical filaments (Figure 2). Co-localization of caspase-cleaved tau was also shown to occur with ubiquitin, one of the primary protein components of CA [9]. Taken together, these results support the notion that CA found in VaD contain truncated and aggregated tau. A recent report in postmortem brain sections from mild cognitive impairment (MCI) subjects confirmed the localization of CA within the hippocampus and there were statistically greater numbers of CA relative to subjects classified as being cognitively normal [28].

Besides our study, Meng et al. also reported the presence of CA in VaD with the demonstration of the presence of two proteins, ADAMTS13 and thrombospondin-1, both highly expressed in blood [17]. The staining of thrombospondin-1 within CA had a halo appearance in some regions, but a more homogenous labeling in other regions [17]. Thrombospondin-1 labeling of CA was found in both normal and VaD cases, but the staining was more frequent and prominent in VaD cases [17]. In addition to thrombospondin-1, the authors also documented the immunoreactivity of the vascular protein, ADAMTS13 in VaD patients. The authors theorized that since the documented CA in VaD were found in perivascular regions, extravasated plasma proteins such as thrombospondin-1 and ADAMTS13, which could leak out of vessels over time, may contribute to the formation of CA [17]. Because VaD is a cerebral vascular disorder, the findings of plasma proteins ADAMTS13 and thrombospondin-1 within CA may not be surprising given the fact the blood-brain barrier is known to be compromised in VaD [48].

## Potential Role of Corpora Amylacea in Vascular Dementia and Other Neurodegenerative Diseases

Although the presence of CA in normal aging and in various neurodegenerative diseases is now well documented, the precise role these structures play has remained elusive. Previous studies have suggested that CA could be important indicators of neurodegeneration. Singhrao et al. proposed that based on the composition of CA, namely the inclusion of ubiquinated proteins and the presence of complement factors that a function of CA could be to prevent the recognition of these immunogenic proteins by immune cells of the CNS and thus protect the CNS from inflammatory injury [12]. Others have supported this view that CA are involved in the sequestration of potentially deleterious cellular products including ubiquitin and heat shock proteins [9,49]. The genesis of CA formation has been correlated to cellular stress, specifically to mitochondrial dysfunction and oxidative stress [50]. The connection with oxidative stress is further supported by data demonstrating the presence of transglutaminase-1 within CA. Transglutaminases are stress-associated enzymes that when activated result in the cross-linking and aggregation of cellular proteins that form the core of the CA [10].

Taken together, the prevailing data suggest that CA serve as protective structures and in this manner share many similar characteristics to Hirano bodies. Hirano bodies were first described in 1965 and are characterized as rod-shaped paracrystalline structures in the neurons of the CNS [51]. Hirano bodies are rich in cytoskelet al. proteins including tau and actin as well as truncated proteins such as TDP-43 [52–54]. Hirano bodies have been found in a number of different neurodegenerative diseases including AD [55], Creutzfeldt-Jacob disease [56], Pick’s disease [51] and Parkinson’s [57]. However, like for CA, whether or Hirano bodies contribute to the neurodegeneration observed in these disorders is unknown.

## Concluding Remarks

The presence of CA within the normal aging brain suggests that these spherical structures are a byproduct of the normal aging process. However, the presence of CA in numerous neurodegenerative diseases and disorders in significant numbers above and beyond what are found in matched controls suggests an alternative role for CA: that they may provide a protective role in these pathological processes by sequestering aggregated protein structures and thus protect cellular structures from further damage. In this manner, CA may play a similar role as to what has been previously described for Hirano bodies and suggest that these structures do not play a passive role but may actually contribute to defense mechanisms that are valiantly attempting to prevent cellular damage in the face of toxic mediators including tau, alpha-synuclein, ubiquitin, and transglutaminases. However, further research is necessary in order to clearly delineate the role of CA in neurodegenerative diseases.
